# Using deep learning to distinguish malignant from benign parotid tumors on plain computed tomography images

**DOI:** 10.3389/fonc.2022.919088

**Published:** 2022-08-01

**Authors:** Ziyang Hu, Baixin Wang, Xiao Pan, Dantong Cao, Antian Gao, Xudong Yang, Ying Chen, Zitong Lin

**Affiliations:** ^1^ Department of Dentomaxillofacial Radiology, Nanjing Stomatological Hospital, Medical School of Nanjing University, Nanjing, China; ^2^ School of Electronic Science and Engineering, Nanjing University, Nanjing, China; ^3^ Department of Oral and Maxillofacial Surgery, Nanjing Stomatological Hospital, Medical School of Nanjing University, Nanjing, China

**Keywords:** deep learning, convolutional neural network, residual neural network, parotid tumor, computed tomography

## Abstract

**Objectives:**

Evaluating the diagnostic efficiency of deep-learning models to distinguish malignant from benign parotid tumors on plain computed tomography (CT) images.

**Materials and methods:**

The CT images of 283 patients with parotid tumors were enrolled and analyzed retrospectively. Of them, 150 were benign and 133 were malignant according to pathology results. A total of 917 regions of interest of parotid tumors were cropped (456 benign and 461 malignant). Three deep-learning networks (ResNet50, VGG16_bn, and DenseNet169) were used for diagnosis (approximately 3:1 for training and testing). The diagnostic efficiencies (accuracy, sensitivity, specificity, and area under the curve [AUC]) of three networks were calculated and compared based on the 917 images. To simulate the process of human diagnosis, a voting model was developed at the end of the networks and the 283 tumors were classified as benign or malignant. Meanwhile, 917 tumor images were classified by two radiologists (A and B) and original CT images were classified by radiologist B. The diagnostic efficiencies of the three deep-learning network models (after voting) and the two radiologists were calculated.

**Results:**

For the 917 CT images, ResNet50 presented high accuracy and sensitivity for diagnosing malignant parotid tumors; the accuracy, sensitivity, specificity, and AUC were 90.8%, 91.3%, 90.4%, and 0.96, respectively. For the 283 tumors, the accuracy, sensitivity, and specificity of ResNet50 (after voting) were 92.3%, 93.5% and 91.2%, respectively.

**Conclusion:**

ResNet50 presented high sensitivity in distinguishing malignant from benign parotid tumors on plain CT images; this made it a promising auxiliary diagnostic method to screen malignant parotid tumors.

## Introduction

Parotid tumor is the most common type of salivary gland tumor. The acinar, ductal, and myoepithelial cells that comprise parotid tissues can give rise to a variety of benign and malignant neoplasms. Pre-operative recognition of malignancy in parotid tumors is useful in that it may alert the surgeon to more stringent attention to the operative margin and hence better tumor clearance (1).

From the clinical aspect, although there are some clues of malignancy-rapid growth, skin fixation, ulceration, facial nerve palsy, pain, or cervical node metastasis, but only 30% malignant parotid tumors present with these features (1, 2). Fine-needle biopsy is helpful in differentiating malignancy; however, it is invasive and more dependent on technical skill and experience to obtain adequate specimens, and the few tissues obtained always could not represent the whole tumor (3–5). Computed tomography (CT), as a commonly used imaging technique, is useful to identify the location and size of parotid tumors. However, benign and malignant parotid tumors always have similar CT features; the sensitivity of CT in identifying malignant tumors is unsatisfactory (6–8).

Recently, deep learning methods, especially convolutional neural network (CNN), have demonstrated effectiveness in image recognition tasks. CNN-based tumor segmentation and classification have been widely used in breast cancer (9), lung cancer (10), liver tumor (11, 12), and nasopharyngeal carcinoma (13). For parotid tumors, Xia et al. and Chang et al. had utilized neural network to differentiating benign and malignant parotid tumors on magnetic resonance imaging (MRI) (14, 15). To date, there were no CNN models based on plain CT images to differentiate benign and malignant parotid tumors. Because of the fatty nature of parotid gland (16), the plain CT images usually could visualize the tumors in parotid gland well and provides abundant texture information of parotid tumors (17). So, in this study, we explored using CNN to diagnose parotid tumors on plain CT images.

Residual neural network (ResNet) is a CNN network proposed in 2015. The framework reformulates the layers as learning residual functions with reference to the layer inputs to obtain deeper networks with higher accuracy (18). The ResNet model could employ the entire image and is capable of retaining image information more completely than many CNN networks. It exhibits high diagnostic efficiency for liver fibrosis staging and lung nodule segmentation (19–22). In this study, the applicability of using ResNet to classify benign and malignant parotid tumor on plain CT images was investigated, and the diagnostic efficiency of it was compared with other two networks and oral radiologists.

## Methods and materials

### Data acquisition

An oral radiologist collected the CT images of patients with parotid tumors in our hospital from 2008 to 2020. The inclusion criteria were as follows: (1) primary parotid tumor; (2) definite pathological diagnosis was available after surgery. (3) The CT images were of good quality, without motion artifacts and foreign body artifacts. The approval from the Ethics Committee of our University was obtained prior to performing this study (NJSH-2022NL-069).

The plain CT images of 283 patients (113 males and 170 females; mean age, 50.5 ± 15.6 years; range, 18–73 years) with parotid tumors were included. Of them, 150 were benign (55 males and 95 females; mean age, 51.7 ± 16.3 years) and 133 were malignant (58 males and 75 females; mean age, 50.3 ± 15.2 years). No statistical difference of age and gender was found between benign and malignant tumor group. The pathological classification of the 283 tumors was showed in **Table 1**.

**Table 1:** The pathological classification of the 283 tumors included.

Benign			
	Pleomorphic adenoma		76
	Warthin tumor		46
	Basal cell adenoma		20
	Other		8
Malignant			
	Adenocarcinoma		
		Mucoepidermoid carcinoma	32
		Pleomorphic adenocarcinoma	19
		Acinar cell carcinoma	15
		Adenoid cystic carcinoma	11
		Other	13
	Lymphoma		15
	Squamous cell carcinoma		11
	Other		17

All patients were performed CT examination before surgery; the parameters of CT were as follows: tube potential: 130 kVp, tube current 56 mA, slice thickness: 3 mm, matrix: 512 × 512, window width: 200 Hounsfield units (Hu), window level: 40 Hu.

### Image processing

**Figure 1:**
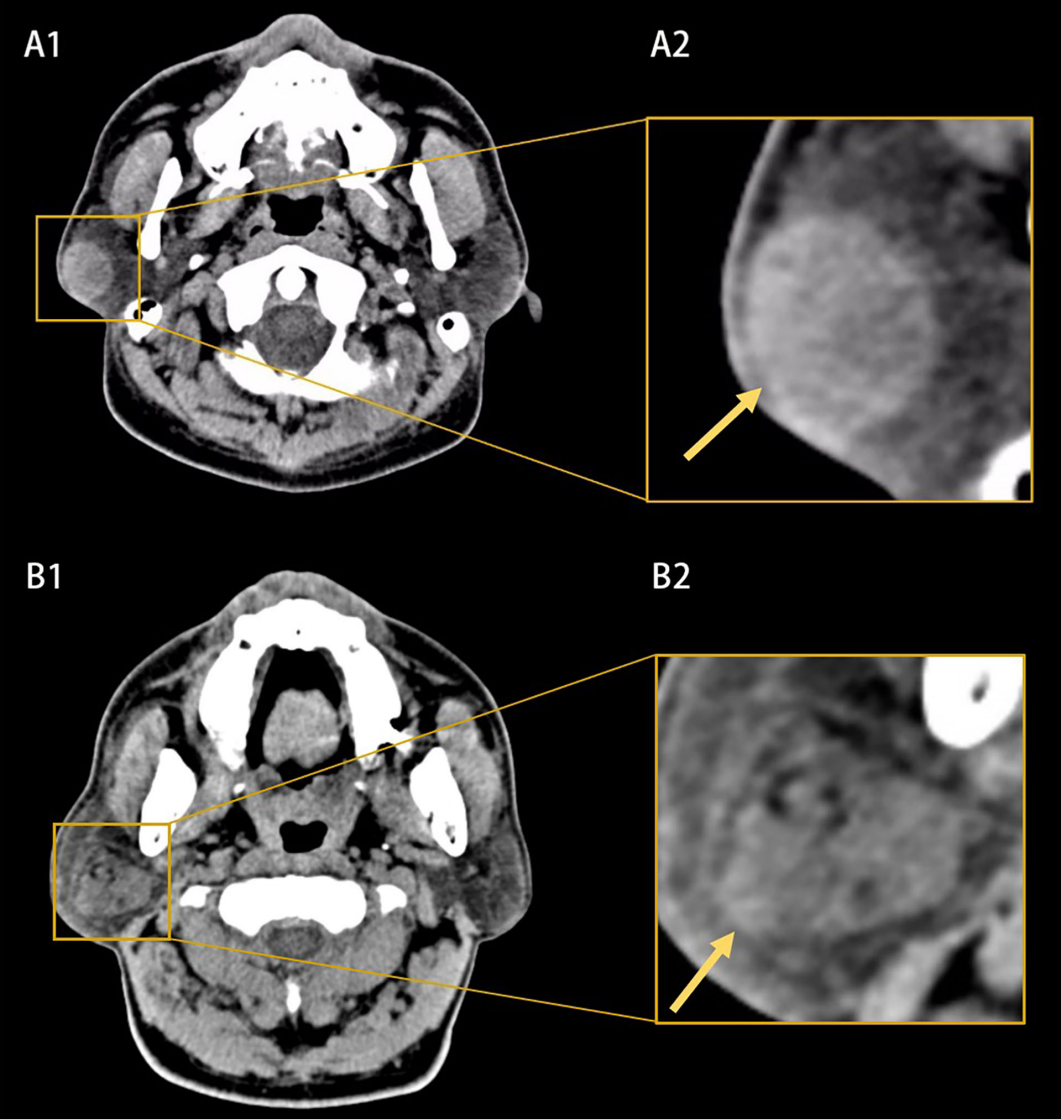
Example of computed tomography (CT) images **(A1, B1)** and the extracted region of interests **(A2, B2)**. **(A1, A2)** Showed a benign parotid tumor with a well-defined and smooth border and homogeneous appearance (yellow arrow). **(B1, B2)** Showed a malignant parotid tumor with a poor-defined border and heterogeneous appearance (yellow arrow).

Two radiologists manually selected the region of interest; axial CT images with lesions were randomly selected and then the regions of interest were obtained by square cropping the CT images (**Figure 1**). For each patient, three or five axial CT images including tumors were selected and cropped. It was confirmed by another radiologist, and if there was any doubt about the area of interest, the two radiologists would work together to re-crop the CT image. Neither of them knew the patients’ pathological diagnosis. As the resolution of images cropped was not equal, the resolution of image was adjusted to a uniform size of 317 × 317 pixels.

**Figure 2:**
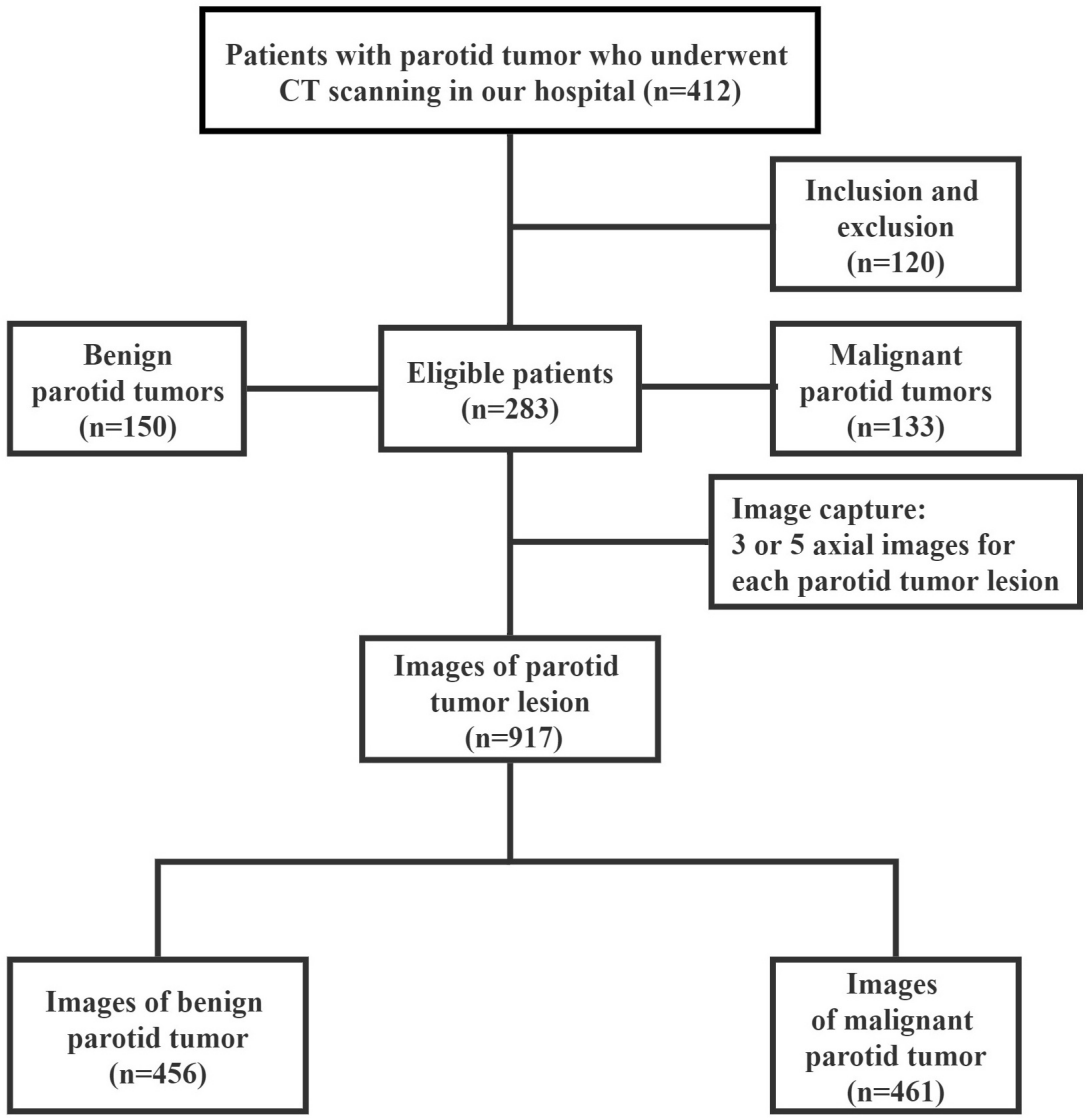
A flowchart of plain computed tomography (CT) image inclusion and exclusion.

A total of 917-cropped CT images were finally obtained (**Figure 2**). The subjects in dataset were divided into two subcategories: training and testing. The training set (approximately 75% of the database [687 images for 213 patients] was used to train variant versions of the model with different initialization conditions and hyper parameters. Once the models have been trained, their performance was evaluated using test set (approximately 25% of the database [230 images for 70 patients]). When building the CNN model, a series of methods were performed on the input images in order to reduce over-fitting of the model. These data argument methods included random horizontal and vertical flipping, random image rotation within 90°.

### Network structure and voting

The CNN models were implemented on hardware with following specification: Intel processor i7, 64 GB RAM with NVIDIA Tesla V100 GPU, 1 TB hard disk for implementing.

**Figure 3:**
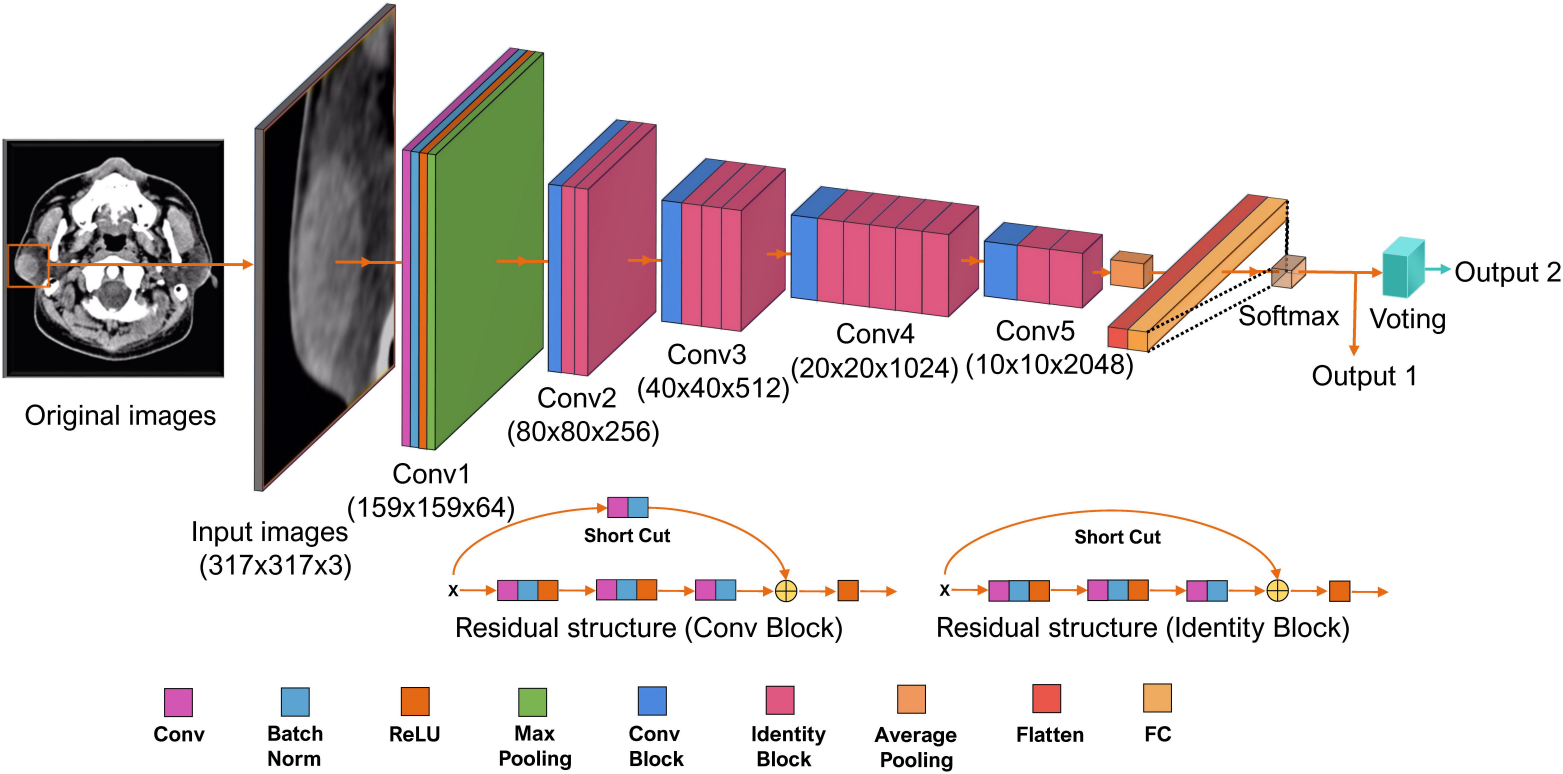
The structure of Residual neural network (ResNet) 50-layer model. The input data are the cropped plain computed tomography (CT) images with a resolution of 317 × 317. It propagated by ResNet through five convolutional phases. Through the five convolutional phases, the data were then processed with three fully connected layers. This ResNet model was structured to output two values; the bigger one indicating the classification of label 0 or label 1 for each CT images is output1. The results of output1 of each CT images are used to perform the final voting. The most labels of output1 for a parotid lesion are output2. Batch Norm is the batch normalization; Conv is the Convolutional layer; FC is the fully connected layer; ReLU is the rectified linear unit function.

ResNet 50-layer structure was shown in **Figure 3** with pre-trained model on the ImageNet database. The input data were grayscale image with a resolution of 317 × 317. The input data were gradually processed by ResNet50 through five blocks. In the first block, the image was converted into a 159 × 159 × 64 tensor. Between the second and fifth stages, a residual block structure was introduced to overcome the problems of vanishing and exploding gradients. After five blocks, the input was converted into a 10 × 10 × 2,048 tensor. Both the height and width were greatly reduced, and the number of dimensions was increased from three to 2,048, which indicated that the extracted information was much more than the original RGB pixel information. According to the 2,048 features extracted by ResNet50, the tensor was fattened into 2,048 vector elements. The model loss function was the cross-entropy loss function and the Random gradient descent (SGD) model optimization method was used. The initial learning rate was 5e−3. The batch size of the model training was 16; the final model selected for the test group was the model with the smallest loss function value for the test group. Fivefold cross-validation was used to establish the ResNet model. The proportion of patients corresponding to benign and malignant parotid tumor was equal for the training and test groups. The final result was the average of the fivefold cross-validation for the test group.

In order to simulate the process of human diagnosis and take the spatial information into account, a voting model was added at the end of Resnet. The input was classified as 0 or 1 (0 and 1 represent benign and malignant parotid tumor, respectively). For each parotid lesion (three or five CT images), the most classification was counted as the final result of the parotid lesion. Generated activation maps by class activation map (CAM) on test dataset were applied to evaluate the region of interest for further clinical review.

Other two neural networks (VGG16_bn and DenseNet169) were used to classify the benign and malignant parotid tumors; the voting model was also added at the end of these networks and the diagnostic efficiency of them was compared with ResNet50. These two networks were also models pre-trained using the ImageNet database (23–25).

### Manual classification of parotid tumor on plain computed tomography images

After development of the CNN models were complete, the 283 parotid tumors were classified into benign or malignant ones by two radiologists (A with 3 and B with 12 years of experience, respectively), using the same CT images. These two observers did not take part in the model training process and were blinded to lesion selection. The observers were also unaware of patient names, laboratory results, other imaging findings, or final diagnosis. After 3 months, observer B re-classified the 283 tumors into malignant or benign; this time, all the original CT images without cropping of the 283 patients were used. The following characteristics were used for classification: tumor location, number of tumors (single or multiple), the size of the tumor (the size based on the selected CT images), tumor shape (regular, e.g., round or oval, irregular, e.g., polycyclic, lobular), tumor density (uniform, uneven), and tumor margins (well defined, poorly defined).

### Statistics

The diagnostic accuracy, sensitivity, specificity, positive predictive value (PPV), and negative predictive value (NPV) of the three CNN networks was calculated on the 917 CT images. The receiver operating characteristic (ROC) curves and the area under the curve (AUC) of the three networks were constructed and calculated. The diagnostic accuracy, sensitivity, specificity, PPV, and NPV; the three deep-learning network models (after voting); and the two radiologists were also calculated for the 283 tumors. The diagnostic accuracy, sensitivity, and specificity of onefold were compared, and the statistical significance was calculated between VGG19_BN, DenseNet169, radiologist A, radiologist B, and radiologist B (second time) with ResNet50 (after voting) using McNemar’s test. The statistical analyses were conducted using SPSS 23.0 software (IBM SPSS Statistics Base Integrated Edition 23, Armonk, NY, USA).

## Results

### Diagnostic performance of three convolutional neural network models and radiologists

**Figure 4:**
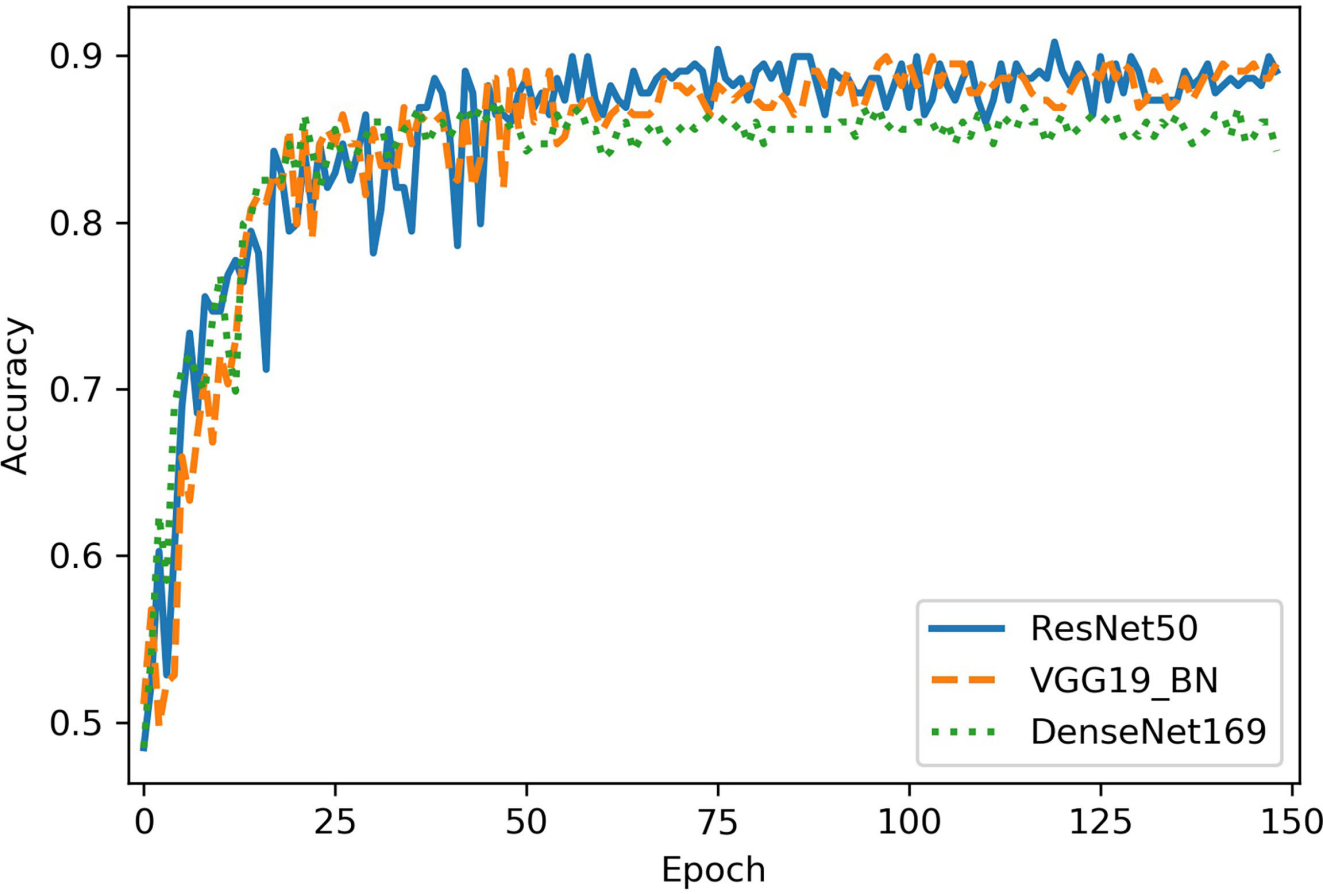
The diagnostic accuracy of Resnet50, VGG16_bn, and DenseNet169 in test set. The horizontal axis represents the training epochs, and vertical axis represents diagnostic accuracy. The best accuracy of ResNet50 is higher than VGG16_bn and DenseNet169.

**Figure 5:**
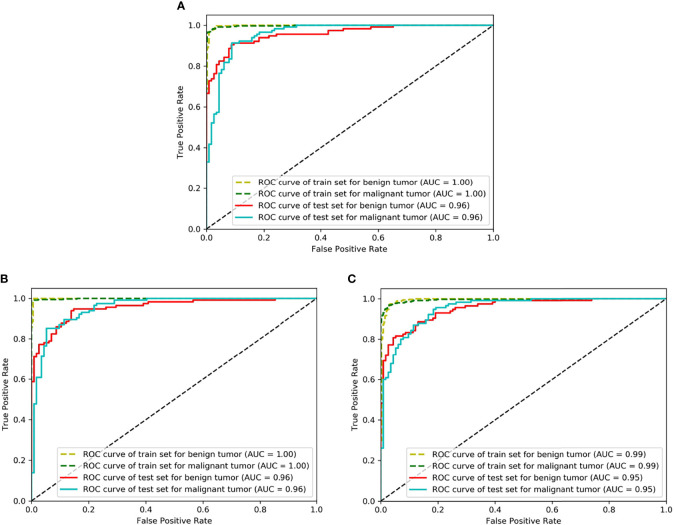
The receiver operating characteristic (ROC) curves of Resnet50, VGG16_bn, and DenseNet169. The horizontal axis represents the false positive rate and vertical axis represents the true positive rate. **(A)** Is the ROC curve of Resnet50 (AUC = 0.96 for malignant tumor), **(B)** Is the ROC curve of VGG16_bn (AUC = 0.96 for malignant tumor), and **(C)** Is the ROC curve of DenseNet169 (AUC = 0.95 for malignant tumor).

The classification performance of three networks was shown in **Table 2**. The accuracy of ResNet50, VGG16_bn, and DenseNet169 was 90.8%, 90.0%, and 87.3%, respectively (**Figure 4**). The ROC curves of the three networks were shown in **Figure 5**. The AUC of Resnet50, VGG16_bn, and DenseNet169 to differentiate malignant from benign tumors was 0.96, 0.96, and 0.95, respectively.

**Table 2:** The diagnostic performance of the three convolutional neural network (CNN) models.

	Accuracy	Sensitivity	Specificity	PPV	NPV
ResNet50	90.8%	91.3%	90.4%	90.5%	91.2%
VGG19_BN	90.0%	85.2%	94.7%	94.2%	86.4%
DenseNet169	87.3%	86.1%	88.6%	88.4%	86.3%

**Figure 6:**
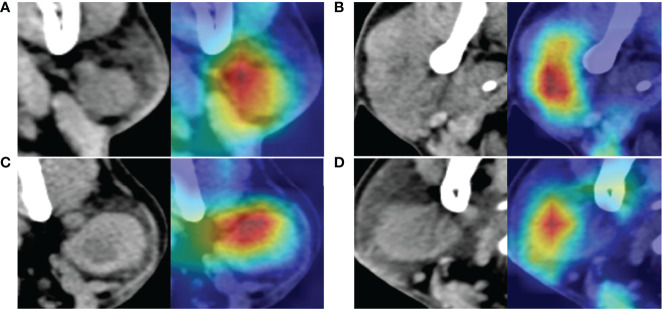
The images at the left side are the original computed tomography (CT) images; the images at the right side are the heatmaps drawn by class activation map (CAM). The red color shows where the network is focused to differentiate benign and malignant parotid tumor. **(A)** Is a benign tumor with homogeneous density and well-defined margin, network mainly focused on the texture and margin of tumor. **(B)** Is a malignant tumor with heterogeneous density and poor-defined margin, network mainly focused on the texture of tumor. **(C)** Is a benign tumor with intra-tumoral cystic component and well-defined margin, whereas network mainly focused on the upper part of the tumor and the margin of the tumor but not the intra-tumoral cystic component. **(D)** Is a malignant tumor with homogeneous density and relative well-defined margin, network mainly focused on texture of the left part of tumor but not the margin of tumor. All these tumors are correctly recognized by the neural network.

The attention heatmap was generated by CAM and then the heatmap was super-imposed on the original CT image, so that the location of parotid tumor and the region highlighted by ResNet could be compared. As showed in **Figure 6**, the attention heatmap highlighted important sub-regions for further clinical review. This showed that the abnormal characteristics of malignant parotid tumors had been learned by Resnet and used as the basis for its classification of benign and malignant tumors.

### Diagnostic performance of different convolutional neural network models after voting

The accuracy, sensitivity and specificity, PPV, and NPV of the three networks after voting and the two radiologists were shown in **Table 3**. Statistical significance between VGG19_BN, DenseNet169, radiologists, and ResNet50 of onefold was shown in **Table 4**.

**Table 3:** The diagnostic performance of three convolutional neural network (CNN) models after voting and two radiologists.

	Accuracy	Sensitivity	Specificity	PPV	NPV
ResNet50	92.3%	93.5%	91.2%	90.6%	93.9%
VGG19_BN	92.3%	87.1%	97.1%	96.4%	89.2%
Densenet169	90.8%	90.3%	91.2%	90.3%	91.2%
Radiologist A	58.6%	32.9%	83.2%	65.3%	53.7%
Radiologist B	68.5%	49.7%	86.6%	78.0%	64.2%
Radiologist B (second time)	77.1%	74.2%	79.7%	76.6%	77.6%

Radiologist B (second time): The classification using all the original computed tomography (CT) images without cropping.

**Table 4:** Statistical significance between VGG19_BN, DenseNet169, radiologists, and ResNet50 of onefold.

	Accuracy (*P*-value)	Sensitivity (*P*-value)	Specificity (*P*-value)
VGG19_BN vs. ResNet50	1.00	0.51	1.00
DenseNet169 vs. ResNet50	1.00	1.00	1.00
Radiologist A vs. ResNet50	0.00	0.00	0.06
Radiologist B vs. ResNet50	0.00	0.00	0.07
Radiologist B (second time) vs. ResNet50	0.04	0.03	0.04

## Discussion

The pre-operative diagnosis of benign and malignant tumors of the parotid gland is of great clinical significance and can have an important impact on surgical planning. Because of the important function of facial nerve, to preserve facial nerve function is a general and important principle in parotid tumors treatments. For benign lesions, local excision or partial parotidectomy is sufficient and every attempt to preserve facial nerve function should be made during surgery; however, for malignant tumors, a total parotidectomy with sacrifice of any part of the nerves overtly involved in tumor is desirable (26). For surgeons, pre-operatively recognition, the malignancy of parotid tumors is urgently hoped to be resolved, since this is helpful for more adequate pre-operative preparation, more appropriate operation (balance between preserving facial nerve function and avoiding recurrence).

Fine-needle aspiration cytology is used for the pre-operative diagnosis, and high specificity was showed by Piccioni et al.’s study and Dhanani et al.’s study (27, 28). However, due to the difficulty of sampling and the heterogeneity of the tumor, the sensitivity of recognition malignancy was not quite satisfactory (sensitivity: 73%–97%, specificity: 83%–97.9%) (5, 27, 28). In a meta-analysis by Schmidt et al., the sensitivity and specificity were 79% and 96% for malignancy (4). The relatively low sensitivity was due to few tissues obtained for diagnosis, so some malignant tumors would be misdiagnosed (false negative). Ultrasound-guided core needle biopsy could obtain an adequate tissue sample for histological evaluation, which allows classification of malignant and benign tumors and tumor grading. The sensitivity and specificity of it was much higher than fine-needle aspiration (29). However, compared with imaging technique, these two pre-operative techniques are invasive and has a risk of infection (15). A summary of the diagnosis results of fine-needle aspiration cytology and core needle biopsy was showed in **Table 5**.

**Table 5:** The diagnostic sensitivity and specificity of different diagnostic methods.

Diagnostic method	Sensitivity	Specificity
Fine-needle biopsy (4)	79%	96%
Core-needle biopsy (29)	98%	94%
Conventional MRI (30)	76%	91%
Plain CT (6–8)	10-50%	85-95%
Ultrasound (elastography) (31)	67%	64%
CT enhanced scan (DL) (32)	96.7% (first group) 76.7% (second group)	98.9% (first group) 78.8% (second group)
Ultrasound (DL) (33)	77%	81%
MRI (DL) (15)	33%-81.7%	87%-94.6%

DL, deep learning.

In clinic, ultrasound, CT, and MRI are widely used in parotid tumors diagnosis (**Table 5**). The CT technique has high spatial resolution and rapid acquisition (34). CT images are useful in defining the anatomic localization, the extent, the density, the border, and delineation of tumors, and they are useful for detecting metastases and lymph nodes. However, it was not reliable in differentiating benign and malignant parotid tumors. Generally speaking, benign lesions reveal a well-defined and smooth border and have a homogeneous appearance (35–37). However, malignant parotid tumors could also display as a homogeneous density mass with well-defined border (6, 38). Recently, CNNs present high efficiency in image processing and classification tasks in many medical fields. Because of textures of parotid tumors differ depending on the underlying histopathological composition, neural network with pixel level of receptive field could extract more detailed image feature than human (39, 40). This supply a non-invasive pre-surgery malignancy identification of parotid tumors based on various images. By providing accurate, consistent, and instant results for the same input image, it could also increase the accuracy of diagnosis and reduce rote manual tasks, helping to simplify clinical workflow integration for radiologist.

In this study, the sensitivity and specificity of ResNet50 in distinguishing malignant from benign tumors were 91.3% and 90.4%, and the sensitivity and specificity reached 93.5% and 91.2% after voting. VGG16_bn presented a sensitivity of 87.1%, and Densenet169 presented a sensitivity of 90.3% after voting. All the three CNN networks presented high sensitivity, and the ResNet50 presented the relatively higher sensitivity. Meanwhile, the two radiologists had a sensitivity of 32.9% and 49.7%, and a specificity of 83.2% and 86.6%. And radiologist B had a sensitivity of 74.2% and a specificity of 79.7% using the whole original CT images without cropping. There were significant differences between radiologists with ResNet50 for diagnostic accuracy and sensitivity (*P* < 0.05). Because radiologist B (second time) had a diagnosis using all the original CT images without cropping, the diagnosis of accuracy and sensitivity increased. Our manual classification results were also similar with previous study (6–8). The inconsistency of manual classification also reflects the instability of manual diagnosis, and manual classification is more dependent on experience of radiologist. Because approximately 80% of parotid tumors are benign (26), this priori experience will make the radiologist more inclined to diagnose parotid tumors as benign. Considering the relatively low sensitivity of fine-needle aspiration cytology and manual classification, we think that the high sensitivity of our ResNet50 could be an important auxiliary diagnosis. For the CNN highly suspected malignant tumors, if the diagnosis of fine needle aspiration cytology is benign, maybe re-sampling, re-evaluation, or consultation of experienced cytologist is needed.

Recently, Chang et al. and Xia et al. also utilized neural network to differentiate benign and malignant parotid tumors on MRI images. In Chang et al.’s study, U-Net model based on MRI images of 85 parotid tumors (60 benign tumors and 25 malignant tumors) was used, and the diagnostic accuracy, sensitivity, and specificity were 71%, 33%, and 87% for malignant tumors. In Xia et al.’s study, a modified ResNet model was developed based on MRI images of 233 parotid tumors (153 benign tumors and 80 adenocarcinoma), and the accuracy, sensitivity, and specificity were 88.2%, 94.6%, and 81.7% for differentiating benign from malignant parotid lesions. And studies using CNN networks based on portal phase CT images (contrast-enhanced CT) and ultrasound images were also published recently (32, 33). In our study, 283 parotid tumors (150 benign tumors and 133 malignant tumors) were used for training and testing. More malignant parotid tumors were included in our database, and a high sensitivity of differentiating malignant from benign parotid lesions was presented. Compared with these CNN studies (**Table 5**), our study had a relatively large sample size, more balanced benign and malignant parotid tumors and relatively high sensitivity of differentiating malignant from benign parotid tumors. We speculate that maybe more malignant parotid samples trained are the reason for high sensitivity of recognition malignant ones in this study.

In this study, in order to simulate the process of human diagnosis, a voting model was built at the end of the three deep-learning network models, and the accuracy, sensitivity, and specificity of the three CNN models were calculated for the 283 tumors. After voting, the three CNN models all showed higher diagnostic efficiency than the models without voting. The pathology diagnosis was a microscopic diagnosis, so the tumors will be diagnosed as malignant if there are malignant cells. However, imaging reflects the macroscopic morphology, so imaging diagnosis is a probabilistic diagnosis and it needs comprehensive analysis. Zhao et al. recently reported a hybrid algorithm, a Bayesian network branch performing probabilistic causal relationship reasoning and a graph convolutional network branch performing more generic relational modeling and reasoning using a feature representation (41). And their hybrid algorithm achieves a much more robust performance than pure neural network architecture.

In this study, we re-analyzed the mis-diagnosed parotid tumors of CNNs. And we found that the accuracy for lymphoma diagnosis was 73.3% (11/15), for ResNet50 (after voting), this was much lower than the whole database. The lymphomas usually appear as homogeneous and sharply demarcated nodes, just like benign tumors; this maybe the reasons of misdiagnosis. Moreover, most mis-diagnosed malignant parotid tumors were small ones; they were homogeneous and well-defined, and similar with benign tumors. So, lymphoma and small tumors are more likely be mis-diagnosed even for CNNs. Furthermore, using the attention heatmap, we can infer which part of the input image is focused on by the neural network. For some tumors, the highlighted areas were on the margins, and for others, the highlighted areas were intra-tumoral, which means that the neural network focused on the texture of images of these tumors (**Figure 6**). Interpretability is increased through the attention heatmap generated. It may provide new ways of thinking in the diagnosis of parotid tumor.

This study still has several limitations. First, the tumor data included need to be further expanded to get a stable result for neural network. Although most of the parotid tumor is included in this study, some types of tumor are still limited for clinical application. Second, there is no auto-segment or automated detection (R-CNN or Yolo) built in the neural networks. A neural network with auto-segmentation or automated detection need to be explored in further study. And networks using a voxel-based domain and the whole CT images without cropping, combining the radiological with clinical findings are also needed to be explored in the future. Third, this study was based on a single center; an external validation study is needed to validate its diagnostic performance and generalizability. Prospective and multi-institutional datasets are also needed in future studies.

## Conclusion

ResNet50 presented high sensitivity in distinguishing malignant from benign parotid tumors on plain CT images, and this made it a promising auxiliary diagnostic method to screen malignant parotid tumors.

## Data availability statement

The data used to support the findings of this study were supplied by Zitong Lin under license and so cannot be made readily available. Requests for access to these data should be made to linzitong_710@163.com.

## Ethics statement

The approval from the Ethics Committee of our University was obtained prior to performing this study (NJSH-2022NL-069). The data are anonymous, and the requirement for written informed consent was therefore waived.

## Author contributions

Z.Y. Hu contributed to writing of this manuscript, neural network modeling, data analysis and visualization. B.X. Wang contributed to neural network modeling and optimization, data analysis and visualization. X. Pan contributed to collection of materials and data curation. D.T. Cao contributed to data analysis. A.T. Gao contributed to collection of materials. X.D. Yang contributed to study design and interpretation of the results. Y. Chen contributed to study design and neural network modeling and optimization. Z.T. Lin contributed to conceptualization, CT image consultation, review and editing of this manuscript, and funding acquisition. All authors gave final approval and agree to be accountable for all aspects of the work.

## Funding

This work was supported by the General project of Jiangsu Commission of Health (M2021077), the Jiangsu Province Medical Association Roentgen Imaging Research and Special Project Funds (SYH-3201150-0007), the Medical Science and Technology Development Foundation (YKK19090), and the Nanjing Clinical Research Center for Oral Diseases (no. 2019060009).

## Conflict of interest

The authors declare that the research was conducted in the absence of any commercial or financial relationships that could be construed as a potential conflict of interest.

## Publisher’s note

All claims expressed in this article are solely those of the authors and do not necessarily represent those of their affiliated organizations, or those of the publisher, the editors and the reviewers. Any product that may be evaluated in this article, or claim that may be made by its manufacturer, is not guaranteed or endorsed by the publisher.

## Abbreviations

CT, Computed tomography, CNN, convolutional neural network, ResNet, residual neural network, SGD, random gradient descent, CAM, class activation map, PPV, positive predictive value, NPV, negative predictive value, ROC, receiver operating characteristic, AUC, area under the curve.

## References

[1] Ettl T Schwarz-Furlan S Gosau M Reichert TE. Salivary gland carcinomas. Oral Maxillofac Surg (2012) 16:267–83. doi: 10.1007/s10006-012-0350-9 22842859

[2] Wong DS. Signs and symptoms of malignant parotid tumours: an objective assessment. J R Coll Surg Edinb (2001) 46:91–5. 11329749

[3] Ozawa N Okamura T Koyama K Nakayama K Kawabe J Shiomi S. Retrospective review: usefulness of a number of imaging modalities including CT, MRI, technetium-99m pertechnetate scintigraphy, gallium-67 scintigraphy and f-18-FDG PET in the differentiation of benign from malignant parotid masses. Radiat Med (2006) 24:41–9. doi: 10.1007/BF02489988 16715661

[4] Schmidt RL Hall BJ Wilson AR Layfield LJ. A systematic review and meta-analysis of the diagnostic accuracy of fine-needle aspiration cytology for parotid gland lesions. Am J Clin Pathol (2011) 136:45–59. doi: 10.1309/AJCPOIE0CZNAT6SQ 21685031

[5] Kristjan GJ Aida A Jahan A. The accuracy of fine-needle aspiration cytology for diagnosis of parotid gland masses: a clinicopathological study of 114 patients. J Appl Oral ence (2016) 24:561–7. doi: 10.1590/1678-775720160214 PMC5161254 28076460

[6] Berg HM Jacobs JB Kaufman D Reede DL. Correlation of fine needle aspiration biopsy and CT scanning of parotid masses. Laryngoscope (1986) 96:1357–62. doi: 10.1288/00005537-198612000-00008 3784740

[7] Whyte AM Byrne JV. A comparison of computed tomography and ultrasound in the assessment of parotid masses. Clin Radiol (1987) 38:339–43. doi: 10.1016/S0009-9260(87)80203-9 3304787

[8] Urquhart A Hutchins LG Berg RL. Preoperative computed tomography scans for parotid tumor evaluation. Laryngoscope (2001) 111:1984–8. doi: 10.1097/00005537-200111000-00022 11801983

[9] Qi X Hu J Zhang L Bai S Yi Z. Automated segmentation of the clinical target volume in the planning CT for breast cancer using deep neural networks. IEEE Trans Cybern (2020) 52(5):3446–3456. doi: 10.1109/TCYB.2020.3012186 32833659

[10] Hamidian S Sahiner B Petrick N Pezeshk A. 3D convolutional neural network for automatic detection of lung nodules in chest CT. Proc SPIE Int Soc Opt Eng (2017), 10134:1013409. doi: 10.1117/12.2255795 28845077 PMC5568782

[11] Yasaka K Akai H Abe O Kiryu S. Deep learning with convolutional neural network for differentiation of liver masses at dynamic contrast-enhanced CT: A preliminary study. Radiology (2018) 286:887–96. doi: 10.1148/radiol.2017170706 29059036

[12] QaW W Sun Z Zhang Yu Li X Ge W Huang Y. SCCNN: A diagnosis method for hepatocellular carcinoma and intrahepatic cholangiocarcinoma based on Siamese cross contrast neural network. IEEE Access (2020) 8:85271–83. doi: 10.1109/ACCESS.2020.2992627

[13] Ma Z Zhou S Wu X Zhang H Yan W Sun S. Nasopharyngeal carcinoma segmentation based on enhanced convolutional neural networks using multi-modal metric learning. Phys Med Biol (2019) 64:025005. doi: 10.1088/1361-6560/aaf5da 30524024

[14] Chang YJ Huang TY Liu YJ Chung HW Juan CJ. Classification of parotid gland tumors by using multimodal MRI and deep learning. NMR Biomed (2021) 34:e4408. doi: 10.1002/nbm.4408 32886955 PMC7757221

[15] Xia X Feng B Wang J Hua Q Yang Y Sheng L. Deep learning for differentiating benign from malignant parotid lesions on MR images. Front Oncol (2021) 11:632104. doi: 10.3389/fonc.2021.632104 34249680 PMC8262843

[16] Howlett DC Kesse KW Hughes DV Sallomi DF. The role of imaging in the evaluation of parotid disease. Clin Radiol (2002) 57:692–701. doi: 10.1053/crad.2001.0865 12169280

[17] Prasad RS. Parotid gland imaging. Otolaryngol Clin North Am (2016) 49:285–312. doi: 10.1016/j.otc.2015.10.003 26902980

[18] He K Zhang X Ren S Sun J. Deep residual learning for image recognition. In: 2016 IEEE conference on computer vision and pattern recognition (CVPR) (2016). PP:770–8. doi: 10.1109/CVPR.2016.90

[19] Liu H Cao H Song E Ma G Xu X Jin R. A cascaded dual-pathway residual network for lung nodule segmentation in CT images. Phys Med (2019) 63:112–21. doi: 10.1016/j.ejmp.2019.06.003 31221402

[20] Li Q Yu B Tian X Cui X Zhang R Guo Q. Deep residual nets model for staging liver fibrosis on plain CT images. Int J Comput Assist Radiol Surg (2020) 15:1399–406. doi: 10.1007/s11548-020-02206-y 32556922

[21] Liu C Liu C Lv F Zhong K Yu H. Breast cancer patient auto-setup using residual neural network for CT-guided therapy. IEEE Access (2020) 8:201666–74. doi: 10.1109/ACCESS.2020.3035809

[22] Liu W Liu X Peng M Chen GQ Liu PH Cui XW. Artificial intelligence for hepatitis evaluation. World J Gastroenterol (2021) 27:5715–26. doi: 10.3748/wjg.v27.i34.5715 PMC8473592 34629796

[23] Simonyan K Zisserman A. Very deep convolutional networks for Large-scale image recognition. Comput Science (2014) 1–1. doi: 10.48550/arXiv.1409.1556

[24] Szegedy C Ioffe S Vanhoucke V Alemi A. Inception-v4. In: Inception-ResNet and the impact of residual connections on learning (2016). AAAI Press (2017) PP:4278–4284. doi: 10.1609/aaai.v31i1.11231

[25] Szegedy C Vanhoucke V Ioffe S Shlens J Wojna Z. Rethinking the inception architecture for computer vision. IEEE (2016). PP:2818–26. doi: 10.1109/CVPR.2016.308

[26] Sood S McGurk M Vaz F. Management of salivary gland tumours: United kingdom national multidisciplinary guidelines. J Laryngol Otol (2016) 130:S142–s149. doi: 10.1017/S0022215116000566 27841127 PMC4873929

[27] Piccioni LO Fabiano B Gemma M Sarandria D Bussi M. Fine-needle aspiration cytology in the diagnosis of parotid lesions. Acta Otorhinolaryngol Ital (2011) 31:1–4. 21808456 PMC3146330

[28] Dhanani R Iftikhar H Awan MS Zahid N Momin SNA. Role of fine needle aspiration cytology in the diagnosis of parotid gland tumors: Analysis of 193 cases. Int Arch Otorhinolaryngol (2020) 24:e508–12. doi: 10.1055/s-0040-1709111 PMC7575377 33101519

[29] Kim HJ Kim JS. Ultrasound-guided core needle biopsy in salivary glands: A meta-analysis. Laryngoscope (2018) 128:118–25. doi: 10.1002/lary.26764 28699165

[30] Liang YY Xu F Guo Y Wang J. Diagnostic accuracy of magnetic resonance imaging techniques for parotid tumors, a systematic review and meta-analysis. Clin Imaging (2018) 52:36–43. doi: 10.1016/j.clinimag.2018.05.026 29908348

[31] Zhang YF Li H Wang XM Cai YF. Sonoelastography for differential diagnosis between malignant and benign parotid lesions: a meta-analysis. Eur Radiol (2019) 29:725–35. doi: 10.1007/s00330-018-5609-6 PMC6302921 29992386

[32] Zhang H Lai H Wang Y Lv X Chen C. Research on the classification of benign and malignant parotid tumors based on transfer learning and a convolutional neural network. IEEE Access (2021), 9:40360–71. doi: 10.1109/ACCESS.2021.3064752

[33] Wang Y Xie W Huang S Feng M Ke X Zhong Z. The diagnostic value of ultrasound-based deep learning in differentiating parotid gland tumors. J Oncol (2022) 2022:8192999. doi: 10.1155/2022/8192999 35602298 PMC9119749

[34] National Center for Health S. Health, united states. In: Health, united states, 2009: With special feature on medical technology. Hyattsville (MD: National Center for Health Statistics (US (2010). 20698070

[35] Isaza M Ikezoe J Morimoto S Takashima S Kadowaki K Takeuchi N. Computed tomography and ultrasonography in parotid tumors. Acta Radiol (1989) 30:11–1. doi: 10.1177/028418518903000103 2536549

[36] Christe A Waldherr C Hallett R Zbaeren P Thoeny H. MR imaging of parotid tumors: typical lesion characteristics in MR imaging improve discrimination between benign and malignant disease. AJNR Am J Neuroradiol (2011) 32:1202–7. doi: 10.3174/ajnr.A2520 PMC7966029 21724574

[37] Kato H Kanematsu M Watanabe H Mizuta K Aoki M. Salivary gland tumors of the parotid gland: CT and MR imaging findings with emphasis on intratumoral cystic components. Neuroradiology (2014) 56:789–95. doi: 10.1007/s00234-014-1386-3 24948426

[38] Golding S. Computed tomography in the diagnosis of parotid gland tumours. Br J Radiol (1982) 55:182–8. doi: 10.1259/0007-1285-55-651-182 7066618

[39] Okahara M Kiyosue H Hori Y Matsumoto A Mori H Yokoyama S. Parotid tumors: MR imaging with pathological correlation. Eur Radiology (2003) 13:L25–33. doi: 10.1007/s00330-003-1999-0 15018162

[40] Sarioglu O Sarioglu FC Akdogan AI Kucuk U Arslan IB Cukurova I. MRI-Based texture analysis to differentiate the most common parotid tumours. Clin Radiol (2020) 75:877.e815–877.e823. doi: 10.1016/j.crad.2020.06.018 32703544

[41] Zhao G Feng Q Chen C Zhou Z Yu Y. Diagnose like a radiologist: Hybrid neuro-probabilistic reasoning for attribute-based medical image diagnosis. IEEE Trans Pattern Anal Mach Intell (2021) PP:1–1. doi: 10.1109/TPAMI.2021.3130759 34822325

